# Human Adipose-Derived Mesenchymal Stem Cells Are Resistant to HBV Infection during Differentiation into Hepatocytes *in Vitro*

**DOI:** 10.3390/ijms15046096

**Published:** 2014-04-10

**Authors:** Ying Wang, Feng Wang, Hongchang Zhao, Xiaohe Zhang, Haiying Chen, Kaiyu Zhang

**Affiliations:** 1 Infection Department, First Hospital of Jilin University, Changchun 130021, Jilin, China; E-Mails: w_ying11@mails.jlu.edu.cn (Y.W.); jdhaiying@gmail.com (H.C.); jdkaiy@gmail.com (K.Z.); 2 Central Laboratory, Hepatobiliary Disease Hospital of Jilin Province, Changchun 130062, Jilin, China; E-Mail: mmxiaohe@gmail.com; 3 Biochemical Laborarory, Changchun Medical Emergency Center, Changchun 130062, Jilin, China; E-Mail: cczhaohc@gmail.com

**Keywords:** hepatitis B, mesenchymal stem cell, adipose tissue, hepatocyte-like cell, glycogen, albumin

## Abstract

The therapeutic methods for chronic hepatitis B are limited. The shortage of organ donors and hepatitis B virus (HBV) reinfection obstruct the clinical application of orthotopic liver transplantation (OLT). In the present study, adipose-derived mesenchymal stem cells (AD-MSCs) and bone marrow-derived mesenchymal stem cells (BM-MSCs) were isolated from chronic hepatitis B patients and characterized for morphology, growth potency, surface phenotype and the differentiation potential. The results showed that both MSCs had adipogenic, osteogenic and neuron differentiation potential, and nearly all MSCs expressed CD105, CD44 and CD29. Compared with AD-MSCs, BM-MSCs of chronic hepatitis B patients proliferated defectively. In addition, the ability of AD-MSCs to differentiate into hepatocyte was evaluated and the susceptibility to HBV infection were assessed. AD-MSCs could differentiate into functional hepatocyte-like cells. These cells express the hepatic-specific markers and have glycogen production and albumin secretion function. AD-MSCs and hepatic differentiation AD-MSCs were not susceptible to infection by HBV *in vitro*. Compared with BM-MSCs, AD-MSCs may be alternative stem cells for chronic hepatitis B patients.

## 1. Introduction

There are about 350 million hepatitis B virus (HBV) carriers in the world. At present the therapeutic methods for chronic hepatitis B are limited [1]. Chronic HBV infection could cause liver cirrhosis and hepatocellular carcinoma. Orthotopic liver transplantation (OLT) remains the only therapeutic option for patient with end-stage liver disease caused by chronic HBV infection, but it is difficult to solve the shortage of organ donors and the HBV reinfection issue [2,3]. Transplantation of stem cells or hepatocytes may partially solve the problem, so new and more accessible extrahepatic human cell sources, especially stem cell sources, are being extensively investigated. The current research focus on mesenchymal stem cells (MSCs). MSCs were found in human bone marrow [4], placenta [5], umbilical cord blood [6], scalp tissue [7], and so on. It can be induced into multiple-type lineages, such as bone, fat, cartilage, neural cells and hepatocytes [8–10]. MSCs from BM have been induced into hepatocytes [11,12] and a phase I trial of transplantation with autologous BM-MSCs has been conducted [13]. However, traditional bone marrow procurement may be distressing to patients and it has been proved difficult to obtain a sufficient amount of autologous adult stem cells. There were studies which demonstrated that BM-MSCs from chronic hepatitis B patients proliferate defectively and decrease expression of growth factor receptors [14]. The treatment effect of autologous transplantation BM-MSCs for chronic hepatitis B was controversial. Therefore, many studies have been investigated to look for other stem cell sources for replacing BM-MSCs.

In the last decade, studies indicated that MSCs can be isolated from adipose tissue [15,16], the so-called adipose-derived mesenchymal stem cells (AD-MSCs). The biological properties of AD-MSCs, including cell surface phenotype, growth kinetics, gene expression and differentiation potential, are similar to BM-MSCs [17,18]. Several studies have reported that AD-MSCs were capable of differentiation into hepatocytes [19,20]. Firstly, research demonstrated that AD-MSCs treated with hepatocyte growth factor (HGF), oncostatin M (OSM) and dimethyl sulfoxide (DMSO) could differentiate into hepatocyte-like cells. These cells expressed hepatocyte-specific markers during differentiation, such as albumin (ALB) and α-fetoprotein (AFP) [21]. In most of the reports on AD-MSCs, the adipose tissues were from non-HBV infection people. There were no reports about biological property of AD-MSCs from chronic hepatitis B patients. In addition, it was not clear whether chronic HBV infection could affect hepatocyte differentiation function of AD-MSCs, and whether AD-MSCs and hepatocyte differentiated from AD-MSCs could be infected by HBV remained unknown.

In the present study, we isolated AD-MSCs from chronic hepatitis B patients and characterized for morphology, growth potency, surface phenotype and the differentiation potential, and compared these with those of BM-MSCs which were isolated from chronic hepatitis B patients. The ability of AD-MSCs to differentiate into hepatocyte was evaluated. In addition, we assessed whether HBV can infect both AD-MSCs and AD-MSC-derived hepatocyte-like cells *in vitro*.

## 2. Results and Discussion

### 2.1. Characterization of Adipose-Derived Mesenchymal Stem Cells (AD-MSCs)

The recent advances of MSCs in cell biology have led to the therapeutic potential for repairing damaged tissues [22]. MSCs possessed the characteristics such as self-renewal, hepatic differentiation potential [23], longevity and immunosuppressive properties [24]. They could be one of the ideal cells for the treatment of liver dysfunction. BM-MSCs could be induced into functional hepatocyte-like cells; however, it proves difficult to obtain sufficient amount of stem cells for autologous transplantation. Compared with other tissue, adipose tissue is a safe and abundant source of large amounts of MSCs in the body [25]. *In vitro* AD-MSCs show stable growth and proliferation in a culture environment, and could be induced into multi-lineaged cells. Many researchers used abdominal or thigh adipose tissue from healthy donors as stem cells sources, and studies on hepatocyte differentiation of AD-MSCs have been reported [26]. However, reports on hepatocyte differentiation of AD-MSCs from chronic hepatitis B patients have not been investigated.

**Figure 1.**
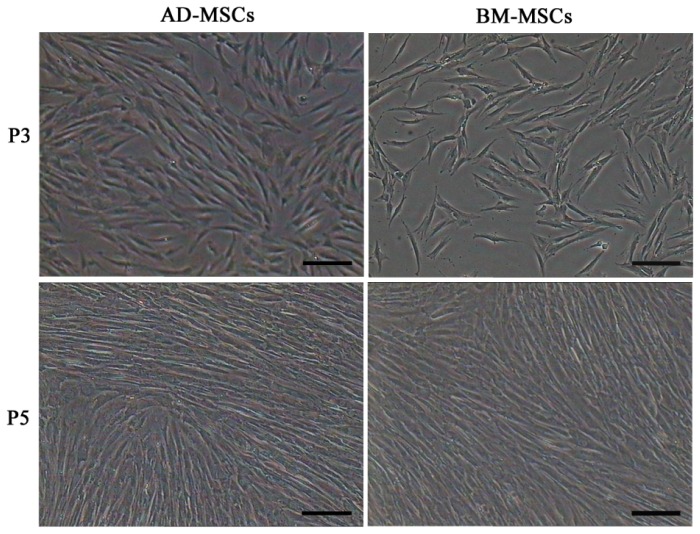
Morphology characteristics of adipose-derived mesenchymal stem cells (AD-MSCs) and bone marrow-derived mesenchymal stem cells (BM-MSCs). Scale bars, 100 μm. (Original magnification, ×100).

Firstly, we isolated AD-MSCs and BM-MSCs from chronic hepatitis B patients, observed their biological characteristics *in vitro* and examined the difference between them. Both AD-MSCs and BM-MSCs from chronic hepatitis B patients could be cultivated and expanded on a plastic dish, and they exhibited a similar fibroblast-like morphology (Figure 1). The success ration of MSCs culture from adipose tissue and BM were 100% (15/15) and 63.6% (7/11), respectively. The primary passage times of MSCs from the two sources were (8.6 ± 1.5) and (16.0 ± 1.9) days, respectively (*p* < 0.05).

**Figure 2.**
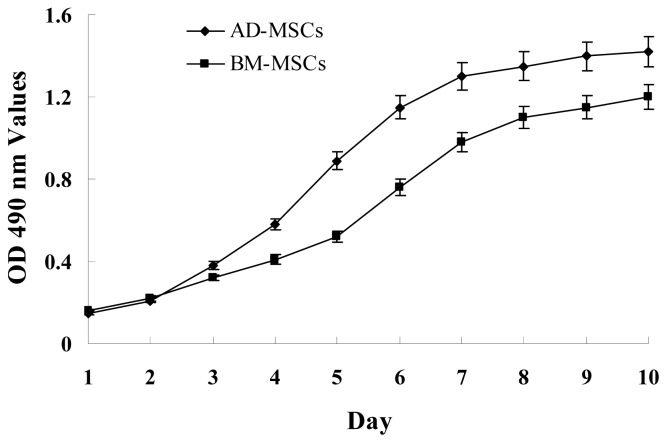
Growth curves of AD-MSCs and BM-MSCs. MSCs were plated at initial density of 2000 cells per cm^2^ and the absorption values were detected over 10 days. Each point on growth curve represents absorption value mean. *n* = 7.

**Figure 3.**
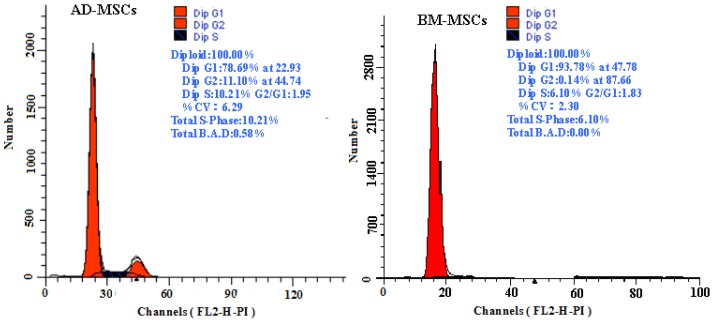
The DNA content of AD-MSCs and BM-MSCs by flow cytometric.

The growth curves of MSCs from the two sources were “S” shape (Figure 2). AD-MSCs came into a logarithmic phase at days 3–4, reached the peak at day 6, and then came into platform at day 7. BM-MSCs came into logarithmic phase at days 4–5, reached the peak at days 8, and then came into platform at day 9. Compared with BM-MSCs, AD-MSCs had shorter incubation time *in vitro*. Furthermore, DNA content of AD-MSCs and BM-MSCs were measured by flow cytometry (Figure 3). The percentage of S-phase nuclei in AD-MSCs was (9.25 ± 1.38)%, which compared with (5.26 ± 1.24)%, the S-phase of BM-MSCs nuclei, the proportion of S-phase AD-MSCs was significantly higher than that of BM-MSCs (*p* < 0.05). BM-MSCs of chronic hepatitis B patients proliferated defectively, which was consistent with the report of Zhong [14] and Fan [27]. Compared with BM-MSCs, AD-MSCs may be alternative stem cells.

**Figure 4.**
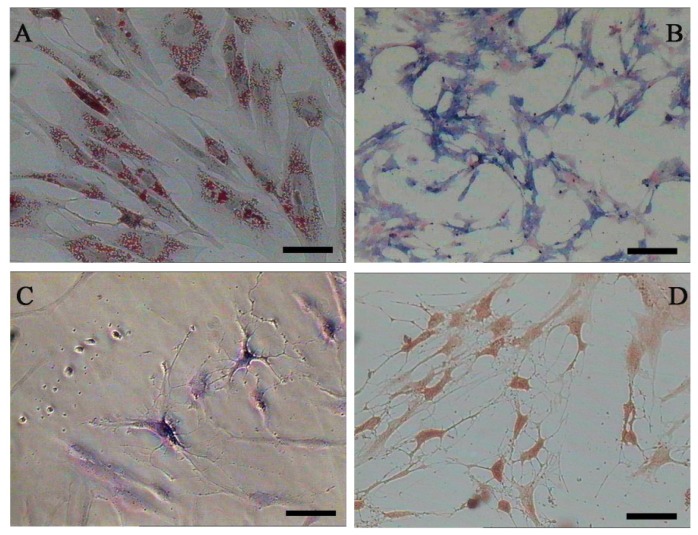
Adipogenic, osteogenic and neurons differentiation of AD-MSCs. (**A**) Oil red O staining positive; (**B**) Alkaline phosphates staining positive; (**C**) Nissl’s body staining positive; and (**D**) Immunocytochemical staining positive for NSE (neuron-specific enolase). Scale bars, 100 μm. (Original magnification, μ100).

**Figure 5.**
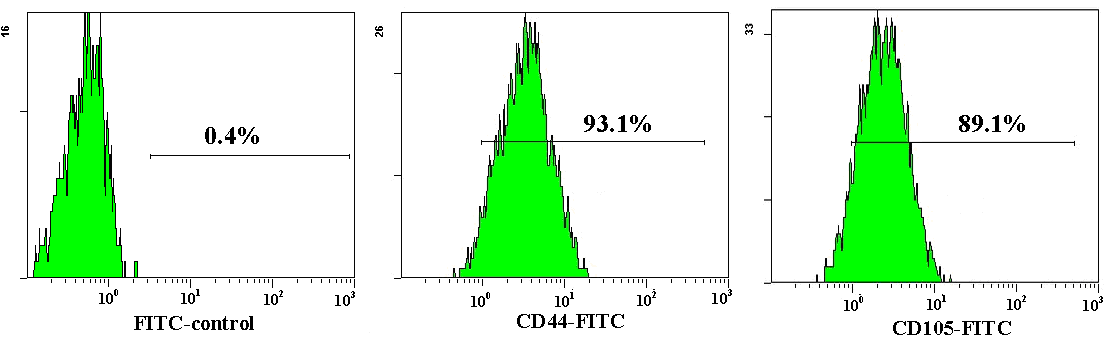

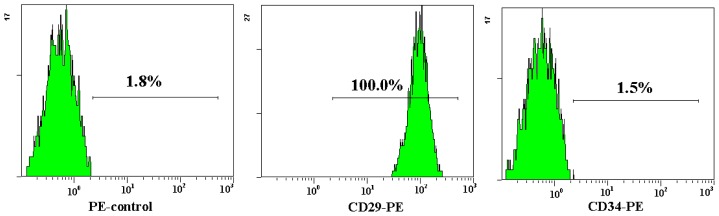
Flow cytometric analysis of AD-MSCs surface antigen. The cell surface marker phenotype of these MSCs was shown to be for CD34−, CD44+, CD105+ and CD29+.

Both AD-MSCs and BM-MSCs from chronic hepatitis B patients have the potential of adipogenic, osteogenic and neurons differentiation, which are consistent with previous reports [4,28] (Figure 4). The surface marker of the 3rd generation AD-MSCs and BM-MSCs were analyzed and the results are consistent with previous report [29]. Nearly all the cells expressed CD44, CD29 and CD105, which are the surface marker characteristics of MSCs. The absence of contaminating hematopoietic cells in the MSCs population was verified by the lack of surface antigen defining hematopoietic progenitor cells (CD34) [26] (Figure 5).

### 2.2. AD-MSCs Differentiated into Functional Hepatocytes

To identify whether chronic HBV infection could affect the hepatic differentiation potential of AD-MSCs from chronic hepatitis B patients, we performed three-step protocol according to a previous report [30], the sequential growth factors, cytokines, hormones, nicotinamide, and DMSO were added to the medium in this protocol. During step I at beginning, AD-MSCs were not greatly changed. However, AD-MSCs gradually changed from fibroblast-like cells to a broad, flattened shape during step II. Finally, the majority of AD-MSCs were changed into hepatocyte-like morphology during step III. The experiment results were in accordance with the report.

**Figure 6.**
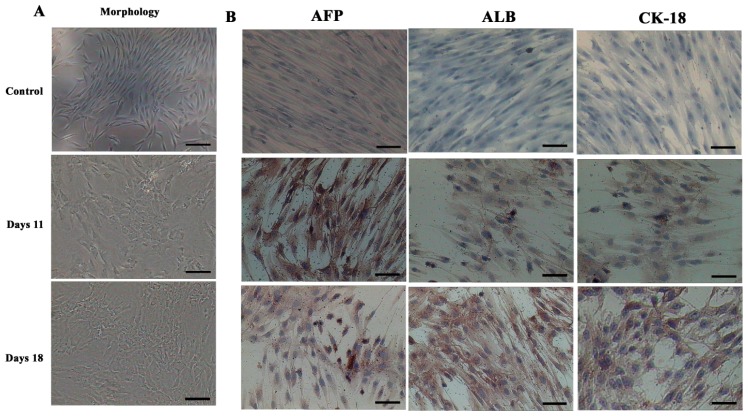
Morphological features and immunocytochemical analysis. (**A**) Morphological features of hepatic differentiated AD-MSCs. The cells’ morphology was gradually changed to polygonal shape at day 11. The majority of the cells were changed into a more polygonal shape at day 18; and (**B**) Immunocytochemical analysis results of the hepatic differentiated AD-MSCs. Differentiated AD-MSCs were intensely stained. Scale bars, 100 μm. (Original magnification, ×100).

**Figure 7.**
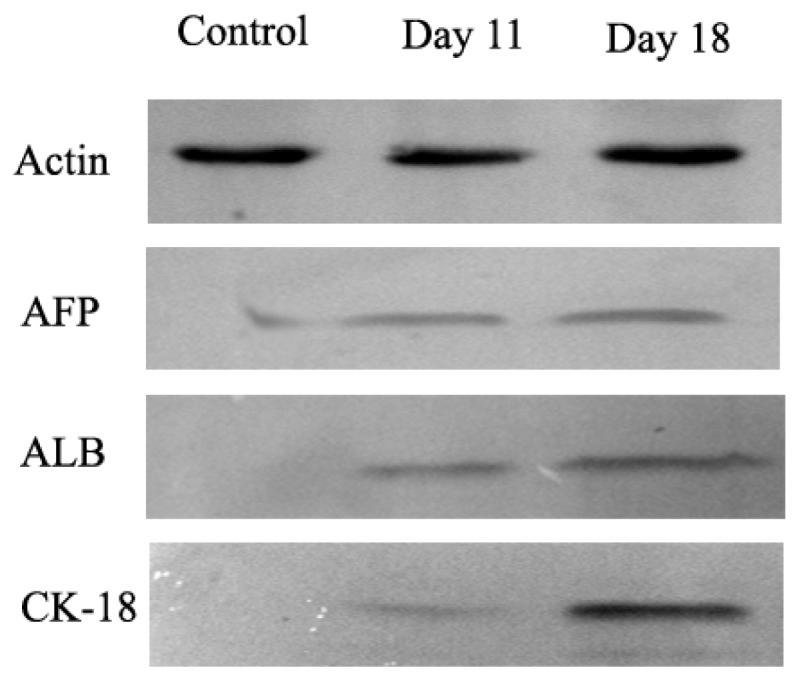
The expression of hepatocyte-specific markers by Western blot. Expression of hepatocyte-specific markers on undifferentiated (Control) and differentiated AD-MSCs at days 11 and 18. Differentiated AD-MSCs expressed or up-regulated protein for α-fetoprotein (AFP), albumin (ALB) and cytokeratin (CK)-18.

In order to determine whether differentiated cells showed the characteristic expression of hepatic-specific markers, we confirmed the expression of AFP, ALB and cytokeratin 18 (CK-18) in differentiated cell populations by immunocytochemical methods and Western blotting at day 11 and 18. The immunocytochemical staining results showed that undifferentiated cells were negative for AFP, ALB and CK-18. AD-MSCs were cultured in accordance with the three-step protocol at day 11 and day 18, the positive rates of AFP were (45.6 ± 6.3)% and (17.6 ± 1.5)%, respectively; the positive rate of ALB were (45.8 ± 5.2)% and (78.9 ± 8.6)%, respectively; the positive rate of CK-18 were (30.5 ± 4.8)% and (70.4 ± 9.3)%, respectively. Compared with the un-induced group, the differences are statistically significant (*p* < 0.05) (Figure 6). The Western blotting results showed the same results as that of immunocytochemical staining (Figure 7). Furthermore, the expression of ALB and CK-18 significantly increased in a time-dependent manner, and AFP expression gradually decreased with increasing induction time. The results demonstrated that AD-MSCs from chronic hepatitis B patients could be induced differentiated into hepatocyte-like cells and gradually matured in the differentiation protocol.

**Figure 8.**
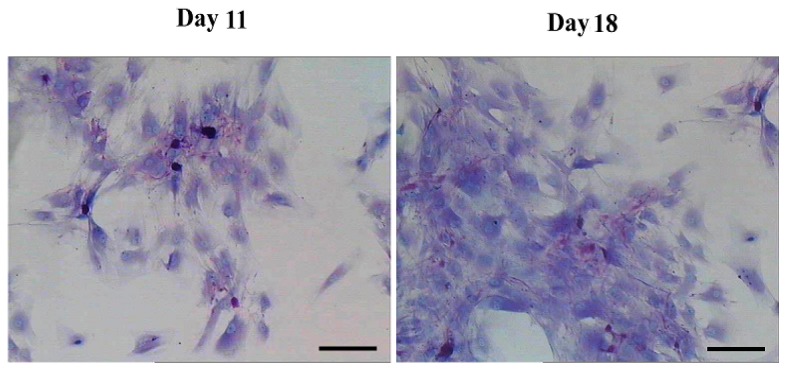
Functional analysis by periodic acid-Schiff (PAS) staining. PAS staining showed positive in AD-MSCs after they were exposed to hepatic differentiation medium at day 11 and day 18. Scale bars, 50 μm. (Original magnification, ×200).

**Figure 9.**
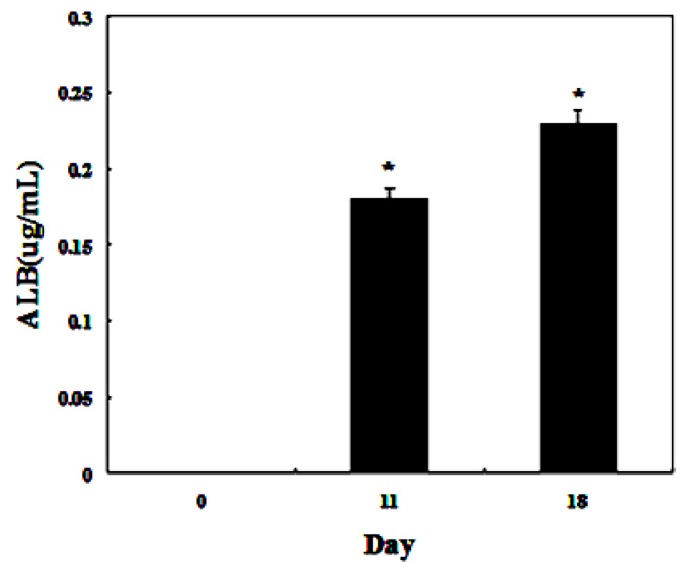
Functional analysis using enzyme-linked immunosorbent assay (ELISA). ELISA analyses showed that differentiated AD-MSCs could secrete albumin. * *p* < 0.05.

To detect whether the differentiated cells possessed mature hepatocyte function, we measured liver functions of the differentiated cells from AD-MSCs. Glycogen production and albumin secretion are unique characteristics of hepatocytes [31]. Firstly we assayed glycogen storage of the differentiated AD-MSCs by periodic acid-Schiff (PAS) staining. AD-MSCs showed no activity of glycogen production in undifferentiated group. Glycogen staining was positive in AD-MSCs after they were exposed to hepatic differentiation medium at days 11 and 18, the positive rate were (41.2 ± 8.5)% and (86.9 ± 11.3)%, respectively (Figure 8). Compared with the un-induced group, the differences are statistically significant (*p* < 0.05). In addition, enzyme-linked immunosorbent assay (ELISA) analyses showed that differentiated AD-MSCs had significantly higher secreted albumin at day 11 (0.18 ± 0.089 μg/mL) and day 18 (0.23 ± 0.098 μg/mL) than undifferentiated AD-MSCs (0.0 ± 0.012 μg/mL) (Figure 9). Compared with the un-induced group, the differences are statistically significant (*p* < 0.05). Glycogen production and albumin secreted significantly increased in a time-dependent manner, with the highest expression level at day 18. The results suggested that AD-MSCs from chronic hepatitis B patients could be induced into functional hepatocyte-like cells *in vitro*.

### 2.3. Hepatitis B Virus (HBV) Failed to Infect AD-MSCs and Hepatic Differentiation AD-MSCs

**Figure 10.**
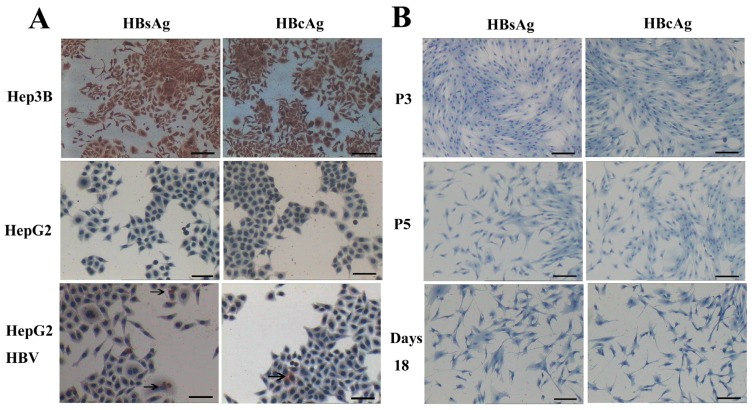
AD-MSCs and AD-MSC-derived hepatocytes are resistant to HBV infection. (**A**) HepG2 cells served as negative control. Hep3B cells were positive control. HepG2 cells infected with HBV (HepG2 HBV) for 48 h were positive for HBsAg and HBcAg; The arrows refer to HBsAg and HBcAg positive cells; and (**B**) The 3rd and 5th generation AD-MSCs (P3, P5) were negative for HBsAg and HBcAg. Induced AD-MSCs at day 18 were negative for HBsAg and HBcAg. Scale bars, 100 μm. (Original magnification, ×100).

**Figure 11.**
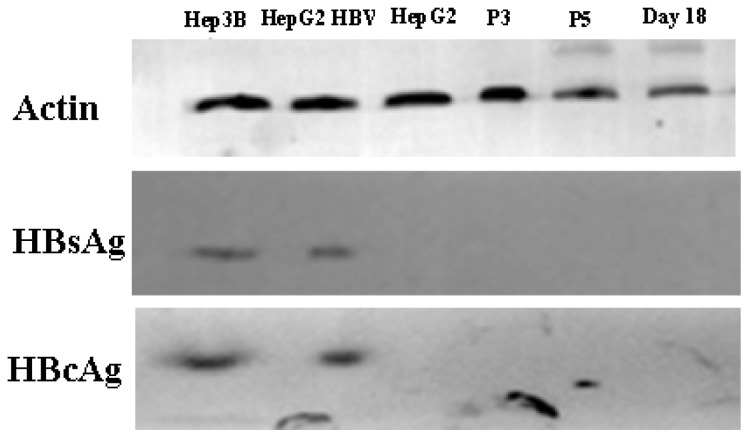
Western blot analysis results. HepG2 cells served as negative controls. Hep3B cells were positive controls. HepG2, the 3rd and 5th generation AD-MSCs and the induced AD-MSCs on day 18 were infected by HBV serum for 48 h. β-actin as control.

HBV can infect many organs except liver. In a previous study, researchers isolated bone marrow MSCs from hepatitis B patients, analyzed the presence of HBV antigens and HBV DNA in BM-MSCs, and their results showed that the MSCs were not susceptible to infection by HBV [32]. However, another study demonstrated that HBV can infect and replicate in cultured human BM-MSCs [33]. It has been unclear whether AD-MSCs from chronic hepatitis B patients could be infected by HBV *in vitro*. We isolated AD-MSCs from chronic hepatitis B patients. The 3rd and 5th generations of AD-MSCs and the hepatic differentiation AD-MSCs at days 11 and 18 were incubated in the HBV infectious environment. HBV antigens were detected by immunocytochemical staining and Western blotting method. The results showed that AD-MSCs and the hepatocyte-like cells from AD-MSCs were not susceptible to infection by HBV (Figures 10 and 11). It may be safe for chronic hepatitis patients to apply AD-MSCs for autologous transplantation.

## 3. Experimental Section

### 3.1. Adipose Tissue and Bone Marrow from Human Subjects

Subcutaneous adipose tissues were resected from 15 chronic hepatitis B patients (age: 35–55; male: 11 and female: 4) during portal vein disconnection. 10 to 50 g of adipose tissues were obtained from each patient. Bone marrow tissue was aspirated from 11 chronic hepatitis B patients (age: 38–57; male: 7 and female: 4) during autologous bone marrow stem cells transplantation. All patients were at Hepatobiliary Disease Hospital of Jilin Province. HBV infection was confirmed via laboratory examination. The detection of HBsAg, HBeAg and HBcAb (Roche Elecsys 2010, Roche, Mannheim, Germany) in serum was positive, and the average HBVDNA copies (Roche COBAS Amplirep/Taqman, Roche, Mannheim, Germany) was 7.5 × 10^5^ IU/mL. The study was approved by the review committee of the hospital, and an informed consent was obtained from each donor.

### 3.2. MSCs Isolation and Culture

AD-MSCs were separated using the method described in a previous report [16]. Resected adipose tissue was minced and then digested in 0.1% collagenase type I (Sigma, Rankonkoma, NY, USA) at 37 °C for 1 h, digested cells were filtered with a cell strainer (BD Bioscience, Franklin Lakes, NJ, USA), and then centrifuged with Ficoll (*d* = 1.077; Pharmacia, Stockholm, Sweden) at 2000 rpm for 20 min; BM-MSCs were separated from 10 mL bone marrow by Ficoll gradient centrifugation method, which was previously described in [4].

The top layer of mononuclear cells were collected and washed with low-glucose DMEM (L-DMEM, Invitrogen, Carlsbad, CA, USA) three times, then the cells were seeded into 50 cm^2^ flasks at a density of 10^6^ cells/cm^2^, and cultured in DMEM with 10% fetal bovine serum (FBS; Hyclone, Logan City, UT, USA) at 37 °C in a 95% humidified incubator with 5% (*v*/*v*) CO_2_. After 72 h of incubation, non-adherent cells were removed. Cell density and morphology were monitored under an inverted microscope (Olympus, Tokyo, Japan). At 80%–90% confluence, the cells were harvested by 0.25% trypsin (Sigma)/0.1% ethylenediaminetetraacetic acid (EDTA) (Sigma), and then subcultured at the density of 1 × 10^4^ cells/cm^2^.

### 3.3. Cell Line

Both HepG2 and Hep3B cells were obtained from the Chinese Academy of Science Type Culture Collection (Shanghai, China) and were cultured in high-glucose DMEM (H-DMEM, Invitrogen) with 10% FBS.

### 3.4. Infection Serum Source

Ten milliliters of vein blood were obtained from chronic hepatitis B patients with HBsAg, HBeAg, HBcAb positive, and the HBV DNA serum load was >10^6^ IU/mL. Vein blood samples were centrifuged at 2000 rpm for 20 min.

### 3.5. MTT Assay for Cells Growth Curve

The 3rd generation of stem cell (Both AD-MSCs and BM-MSCs) was harvested and adjusted the cells concentration to 1 × 10^7^/L. The cells were seeded in a 96-well plate (1 × 10^3^ cells/well). After 24 h, thiazolyl blue tetrazolium bromide (MTT, Sigma) was added to one 96-well plate every day at the same time and incubated for an additional 4 h. The absorption of formazan solubilized in 100 μL of DMSO was measured at the wavelength of 490 nm by a 96-well multiscanner autoreader (Biotech Instrument, Randolph, NJ, USA). There were control holes in each 96-well plate and the cells were cultured continuously for 10 days. The growth curve was drawn with time as abscissa, and the absorbance value as ordinate.

### 3.6. Analysis of MSCs DNA Content

The DNA content of the 3rd generation of stem cells was determined using DNA Reagent Kit (KeyGEN Biotech, Nanjing, China). Briefly, the cells density was adjusted to 1.0 × 10^6^ cells/mL, and then immediately stained according to the manufacturer’s instructions for flow cytometric analysis (BD Bioscience). The data was analyzed using Modifit software (BD Bioscience).

### 3.7. Flow Cytometric Analysis Cell Surface Phenotype

The 3rd generation of stem cells was harvested, and washed once with phosphate-buffered saline(PBS). Cells (1 × 10^5^ per sample) were stained with the following mouse anti-human monoclonal antibody at room temperature for 30 min: anti-CD44-FITC, -CD105-FITC, -CD29-PE, -CD34-PE (Biolegend, San Diego, CA, USA), isotype-identical antibody (Biolegend) served as controls. The cells were washed with PBS two times, and then resuspended in PBS. Fluorescent labeling was analyzed by flow cytometric analysis and CellQuest Pro software (BD Bioscience, Franklin Lakes, NJ, USA).

### 3.8. Differentiation of MSCs into Adipocyt, Osteoblasts and Neurons

The 3rd generation of stem cells was seeded into 12-well plates (1 × 10^5^ cells/well). When 80% confluent, the cells were induced into adipocyte, osteocyte and neurons. Adipogenic differentiation was induced by cultured in adipogenic medium (Zen-Bio, Research Triangle Park, NC, USA) and evaluated by oil red O (Baso, Zhuhai, China) stained at day 14. Osteogenic differentiation was induced by cultured the cells in L-DMED containing 10% FBS, 10 nM dexamethasone, 50 mg/dL ascorbic acid 2-phosphate and 10 mM β-glycerphosphate (Sigma) [34]. Osteogenic differentiation was assessed using alkaline phosphatase staining (Baso) at day 14. Neuron differentiation was induced by β-ME (β**-**mercaptoethanol) and DMSO [4], and evaluated by morphology change, Nissl’s body staining and immunocytochemical staining for neuron-specific enolase (NSE).

### 3.9. Hepatic Differentiation Protocol

AD-MSCs were induced into hepatocyte using the three-step protocol which was described in a previous report [30]. The 3rd generation of AD-MSCs was used for hepatic differentiation assay. The hepatic differentiation potential was assessed at different time-points. The protocol was applied to AD-MSCs from 10 chronic hepatitis B patients.

### 3.10. AD-MSCs and Hepatocyte-Like Cells Derived from AD-MSCs HBV Infection

The method was in accordance with the previous report [24]. The 3rd of AD-MSCs and the AD-MSC-derived hepatocyte-like cells at days 11 and 18 were seeded on cover slips. FBS was deprived of medium after 24 h, and then the cells were incubated with L-DMEM with 2% DMSO (Invitrogen) and 5% HBV sera concentration. After 48 h, the cells were rinsed 5 times with PBS, and then cultured with complete media. Hep3B and HepG2 cells were used as control.

### 3.11. Immunocytochemical Analysis

Immunocytochemical staining was done using the standard protocols. Cultured cells were fixed in 4% paraformaldehyde (Dingguo Biotech, Beijing, China) for 20 min and rinsed with PBS for three times. They were permeabilized with 0.5% Triton X-100 (Dingguo Biotech) for 10 min at room temperature, and then incubated in 3% hydrogen peroxide (Dingguo Biotech) for 30 min to quench the endogenous peroxidase activities. The cells were incubated with mouse monoclonal anti-albumin (1:100, Dako, Glostrup, Denmark), anti-AFP (1:100, Dako), anti-CK-18 (1:200, Dako), anti-HBsAg (1:100, Biolegend), anti-HBcAg (1:100, Biolegend) for 4 h at 37 °C, and then rinsed with PBS five times. They were incubated with anti-mouse IgG (1:1000, Biolegend) for 30 min. Immunoreactivity was visualized utilizing 3,3-diaminobenzidine tetrahydrochloride (DAB, Zhongshanjinjiao Biotech, Beijing, China) and counterstained with hematoxylin (HE, Baso) for 5 min, and then observed under a light microscope (Olympus).

### 3.12. Western Blotting Analysis

Western blotting method was in accordance with the standard protocols. We used mouse monoclonal anti-albumin (1:100), anti-AFP (1:200), anti-CK-18 (1:100), anti-HBsAg (1:100) anti-HBcAg (1:100) and anti-β-actin (1:500, Dako). We used goat antibodies to mouse IgG (1:500) and developed the blots by DAB.

### 3.13. Enzyme-Linked Immunosorbent Assay (ELISA) for Albumin Secretion

The human albumin content in the supernatant was detected using the Human albumin ELISA quantitation kit (Belthy, Montgomery, AL, USA) according to the manufacture instructions. After 18 days of culture, the cells was transferred to starvation medium (without FBS), and then the supernatant was harvested 24 h later. Samples were analyzed in duplicate under each condition.

### 3.14. Periodic Acid-Schiff (PAS) Staining for Glycogen Deposits

The glycogen deposit in the cells was detected using Periodic acid-Schiff staining kit (Baso) according to the manufacturer instructions. Cultured cells were fixed in 4% paraformaldehyde for 20 min and rinsed with PBS three times. The cells were then oxidized in periodic acid for 15 min at room temperature and washed with deionized H_2_O (dH_2_O). After treating with Schiff solution for 10 min, the cells were counterstained with HE for 2 min, and then rinsed with dH_2_O three times. The purple staining indicated that the presence of glycogen in the cells and were observed using a light microscope.

### 3.15. Statistical Analysis

All data were presented as mean ± SD value. For statistical analyses, we used the *t*-test to compared data collected from AD-MSCs and that of obtained from BM-MSCs, and a non-parametric test to compare the expression of hepatic-specific markers. Significance level of *p* < 0.05 was considered statistically significant.

## 4. Conclusions

The present study indicates AD-MSCs from chronic hepatitis B patients have a similar differentiation potential towards the hepatic lineage as BM-MSCs, but their abundance, accessibility and higher proliferation capacity differ from BM-MSCs. Under certain defined inducing conditions, they can differentiate toward a hepatic phenotype *in vitro* and have hepatic biochemical functions. In addition, AD-MSCs and hepatic differentiated AD-MSCs were not susceptible to infection by HBV. Therefore, adipose tissue seems to be an ideal source of high large amounts of autologous multilineage MSCs for cell therapy of liver dysfunction for chronic hepatitis B patients.
